# Metabolically driven flows enable exponential growth in macroscopic multicellular yeast

**DOI:** 10.1126/sciadv.adr6399

**Published:** 2025-06-20

**Authors:** Nishant Narayanasamy, Emma Bingham, Tanner Fadero, G. Ozan Bozdag, William C. Ratcliff, Peter Yunker, Shashi Thutupalli

**Affiliations:** ^1^ Simons Centre for the Study of Living Machines, National Centre for Biological Sciences (TIFR), Bangalore, India.; ^2^ School of Physics, Georgia Institute of Technology, Atlanta, GA, USA.; ^3^ Interdisciplinary Graduate Program in Quantitative Biosciences, Georgia Institute of Technology, Atlanta, GA, USA.; ^4^ Woods Hole Marine Biological Laboratory, Woods Hole, MA, USA.; ^5^ School of Biological Sciences, Georgia Institute of Technology, Atlanta, GA, USA.; ^6^ International Centre for Theoretical Sciences (TIFR), Bangalore, India.

## Abstract

The ecological and evolutionary success of multicellular lineages stems substantially from their increased size relative to unicellular ancestors. However, large size poses biophysical challenges, especially regarding nutrient transport: These constraints are typically overcome through multicellular innovations. Here, we show that an emergent biophysical mechanism—spontaneous fluid flows arising from metabolically generated density gradients—can alleviate constraints on nutrient transport, enabling exponential growth in nascent multicellular clusters of yeast lacking any multicellular adaptations for nutrient transport or fluid flow. Beyond a threshold size, the metabolic activity of experimentally evolved snowflake yeast clusters drives large-scale fluid flows that transport nutrients throughout the cluster at speeds comparable to those generated by ciliary actuation in extant multicellular organisms. These flows support exponential growth at macroscopic sizes that theory predicts should be diffusion limited. This demonstrates how simple physical mechanisms can act as a “biophysical scaffold” to support the evolution of multicellularity by opening up phenotypic possibilities before genetically encoded innovations.

## Teaser

Metabolically generated flow is an emergent mechanism that alleviates diffusion limits in macroscopic multicellular yeast.

## INTRODUCTION

The evolution of multicellularity transformed life on Earth, evolving repeatedly across the tree of life (*1*–*3*). Size plays a central role in the early evolution of multicellularity, underpinning diverse benefits that favor a multicellular life history. For example, larger size can enable organisms to escape predation by filter feeders, increase resource utilization efficiency, and improve motility (*4*). Within more complex lineages of multicellular eukaryotes (i.e., plants, animals, fungi, and macroalgae), whose success over the past billion years has radically transformed Earth’s ecology, size plays a fundamental role in their life histories, both facilitating extensive ecological niche partitioning and underpinning the evolution of cellular and tissue-level differentiation (*2*, *5*–*8*).

The evolution of large multicellular size, however, poses a number of constraints, many of which are biophysical in nature. One of the most substantial of these challenges is transporting nutrients into (and waste out of) the multicellular group. Beyond a critical size, diffusion alone is unable to transport enough resources to meet the demands of an entire group of cells (*6*, *9*–*11*). As a result, growth is typically confined to the group’s surface and, in general, the biomass increase is not exponential (*12*, *13*). For instance, it is well-known that microorganismal colonies exhibit subexponential growth beyond a certain size due to nutrient or oxygen limitations (*14*–*18*). Further, the absence of nutrients can affect morphology, as seen in bacterial colonies grown in various environments (*19*–*22*). These biophysical constraints may be mitigated by the evolution of de novo biological mechanisms, such as cilia that generate fluid flows to enhance nutrient transport (*9*, *23*), or vascular networks that enable active transport of resources throughout the body (*5*, *11*, *24*–*26*). The evolution of enhanced multicellular transport mechanisms can lead to an evolutionary feedback loop, where the evolution of larger organism size creates steeper diffusive nutrient gradients, which favors the evolution of increasingly sophisticated transport mechanisms, thereby allowing body size to further increase. This positive feedback is thought to play a fundamental role in the evolution of large, complex multicellular organisms such as plants and animals (*11*). While both theoretical predictions (*6*, *9*–*11*, *14*, *15*, *27*) and experiments with extant organisms (*10*, *28*, *29*) suggest that diffusion limitation is an unavoidable constraint on early multicellularity, it is difficult to address this question directly, as the early ancestors of extant multicellular lineages have long been extinct.

Using long-term experimental evolution of multicellularity in the snowflake yeast model system, we demonstrate that an emergent biophysical mechanism can overcome transport constraints, enabling exponential growth even at macroscopic sizes beyond diffusive transport limits. Snowflake yeast have been undergoing selection for large size in the multicellularity long-term evolution experiment (MuLTEE) for more than 1000 daily rounds of selection (*4*, *17*, *30*–*33*). Within the first 600 days, they evolve macroscopic group size, where individual clusters are millimeters in diameter, and contain hundreds of thousands of clonally related cells (*30*). We show that, beyond a threshold cluster size, the metabolic activity of snowflake yeast causes spontaneous buoyancy-driven flows through the cluster that actively transport nutrients, sustaining exponential growth well beyond prior theoretical predictions. This work demonstrates how simple physical processes can act as biophysical scaffolds, opening up frontiers of phenotypic evolution in nascent multicellular organisms even before the evolution of genetically encoded innovations.

## RESULTS

**Fig. 1.**
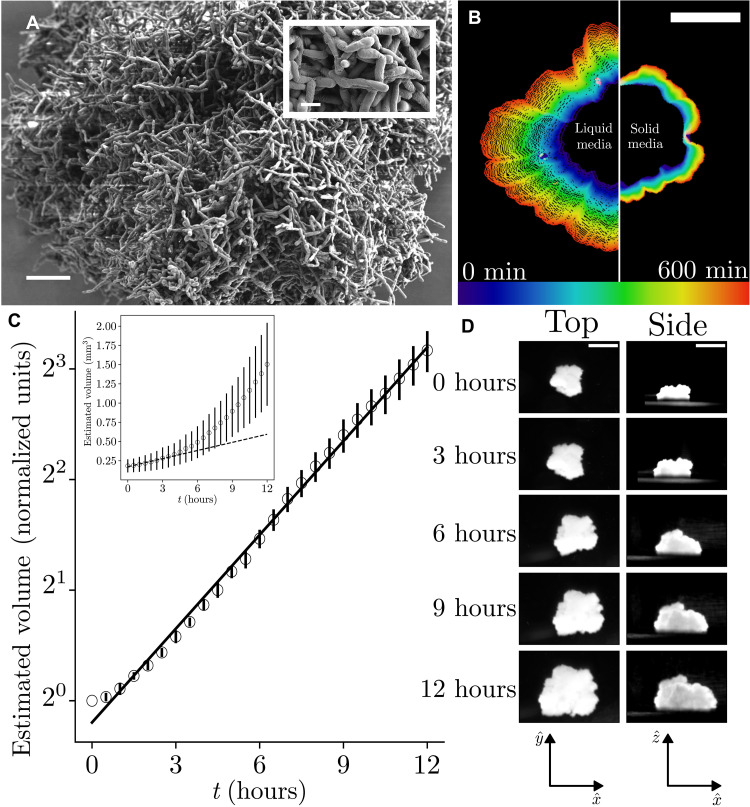
Fluid environments allow for exponential growth of the snowflake yeast clusters. (**A**) Scanning electron microscope image of a snowflake yeast cluster. Scale bar, 20 μm. Inset: Higher-resolution image of single cells within the cluster. Scale bar, 5 μm. (**B**) Cluster outline visualized as a function of time in nondeformable [yeast extract peptone dextrose (YEPD) agar] medium and fluid (YEPD liquid) environment for a single measurement over 60 min with a 10-min interval. (**C**) Quantification of different growth phenotypes in deformable (*N* = 8) versus nondeformable (*N* = 10) media. Scale bar, 1 mm (details in the Supplementary Materials). Estimated volume (area of top view times average height of side view) of snowflake yeast clusters over time, normalized to that at the first time point. Error bars represent SE of 3 replicates. Inset: Estimated volume, without normalization. The dashed line shows a linear fit to the first 3.5 hours. (**D**) Microscopy images of the top and side view over time of one of the clusters measured for (C). Scale bars, 1 mm.

Snowflake yeast clusters grow due to the proliferation of their constituent *Saccharomyces cerevisiae* cells, which undergo incomplete cell division. This causes daughter cells to remain attached to parents, thereby forming cellular chains within the clusters. Snowflake yeast remain mechanically stable even at macroscopic sizes due to the entanglement (*31*) of the cellular chains within the groups (Fig. 1A). These large clusters far exceed the size (≈50 μm) at which diffusive transport alone is predicted to be sufficient to meet the cellular growth demands (*18*, *22*, *34*–*36*) (Supplementary Materials). Further, yeast cells do not have flagella or cilia, and snowflake yeast have no known multicellular adaptations that would allow the active transport of nutrients (Fig. 1A, inset). The growth of these large clusters was therefore expected to be limited by the diffusive transport of nutrients deep into highly entangled clusters and thus subexponential. However, we found that in nonagitated nutrient liquid media, macroscopic snowflake yeast exhibits much faster and competitive overgrowth in contrast to growth on solid agar substrates (Fig. 1B and figs. S1 to S6 and movies S1 and S2). We find that the growth of the macroscopic clusters immersed in a fluid environment remains exponential, even at millimetric sizes, while the growth on solid substrates becomes subexponential (linear) (Fig. 1C, figs. S6 and S7, and movies S1 and S2).

**Fig. 2.**
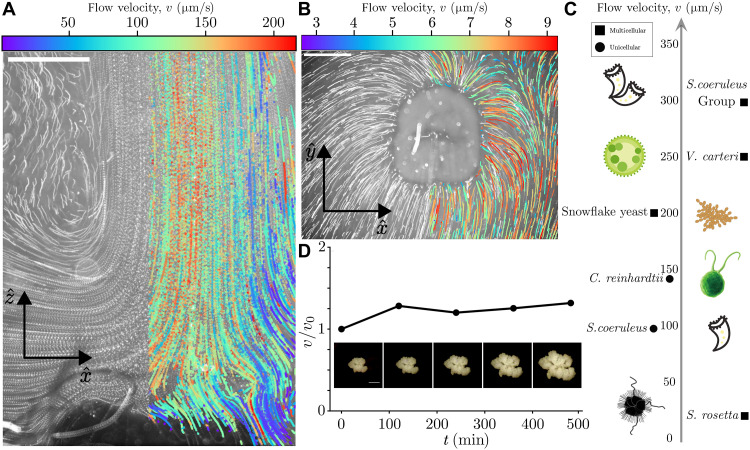
Macroscopic snowflake yeast advectively mixes its ambient fluid environment. Macroscopic snowflake yeast generate three-dimensional flows in the ambient fluid as visualized by tracer particle streaks and particle tracking data from a (**A**) side view and a (**B**) top view. Scale bar, 500 μm. (**C**) Flow speeds are comparable to those generated by ciliated and flagellated multicellular organisms: *S. coeruleus* (1 to 2 mm), *Volvox carteri* (0.5 to 2 mm) *Chlamydomonas reinhardtii* (10 to 20 μm), and *Synaptula rosetta* colony (10 to 30 μm). Graphics are obtained from the Database Center for Life Sciences (CC BY-SA 4.0). (**D**) Flow remains constant over a period of nearly 500 min during which the growth measurements were made (single observation).

These observations led us to ask how liquid media could support exponential growth of the macroscopic snowflake yeast. Given that the limits of diffusive transport were bypassed, we hypothesized the presence of an advective rather than diffusive fluid environment, which could transport nutrients deep into the cluster. We found strong three-dimensional flows around the snowflake yeast (Fig. 2, A and B; figs. S8 and S9; and movies S3 and S4). These flows had a stereotypic circulatory structure: The fluid enters from the sides of the cluster and exits from the top. The fastest flows around the snowflake yeast are comparable to flow speeds generated by other similarly sized multicellular ciliated and flagellated organisms such as *Volvox* (*37*, *38*), choanoflagellates (*39*–*41*), *Chlamydomonas* (*37*, *42*), and colonial stentors (*43*) (Fig. 2C). (Figure 2C only includes measurements of fluid flows, not swimming speeds. Some organisms, such as *Choanoeca flexa*, are able to swim at similar speeds (*44*).) These flows persist with relatively constant speeds throughout the growth of the snowflake yeast cluster (Fig. 2D).

While investigating the mechanism behind the flows, two observations stood out as potentially important. First, as described above, flows have a stereotyped orientation—fluid moved in at the cluster sides and upward from the top of the cluster, and flows are centered on the cluster. Second, the snowflake yeast clusters we studied rely on fermentation alone for their metabolism, so that in addition to depleting the surrounding media of glucose, they also produce ethanol and CO_2_, all of which are less dense than the glucose-rich media. This observation suggests that the flows could be due to the generation of mass density gradients in the fluid. Previous work has shown that microbial colonies can generate flows in their surrounding fluid due to these density gradients (*45*, *46*). We ruled out other possibilities such as Marangoni flows, driven by surface tension gradients (*47*), and evaporation-driven fluid currents (*48*) (see the Supplementary Materials for more details). Based on these observations, we hypothesized that the flows we observed were due to spontaneously generated fluid mass density gradients driven by the metabolic activity of the clusters.

**Fig. 3.**
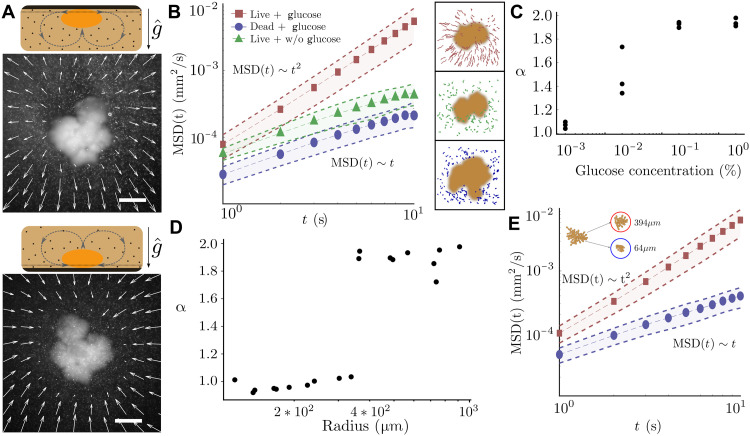
Snowflake yeast beyond a threshold size drive buoyant flows due to their metabolic activity. (**A**) Flows around snowflake yeast immobilized in agar (black region). The dotted line shows the imaging plane in which the flows are measured. The top and bottom panels show the flows in the same imaging plane when the experimental setup is flipped with respect to the direction of gravity. Scale bar, 500 μm. Reversal of flow in the same imaging plane hints at a gravity sensitive flow mechanism such as buoyant flows. (**B**) Metabolic activity is necessary for the flows, which are quantified by the MSD of tracer beads around the snowflake yeast. Tracer beads around live, metabolically active snowflake yeast exhibit ballistic motion i.e., α ≈ 2 (square data points), while tracer beads diffuse, i.e., α ≈ 1, around metabolically inactive and dead clusters (triangles, circles respectively). (**C**) Metabolically active flows emerge at high enough glucose concentration in the ambient medium. (**D**) Flows emerge around clusters beyond a certain threshold size along the evolutionary lineage of the MuLTEE. The exponent α of the tracer particle MSDs exhibits a clear and sharp transition from a diffusive behavior (i.e., α ≈ 1) to a ballistic behavior (i.e., α ≈ 2). (**E**) When clusters large enough to produce flows are broken to sizes below the threshold size identified in (D), the clusters no longer generate flows (α ~ 1, circles). On the other hand, clusters that are individually below the threshold size do create a flow when aggregated together to form a larger group (α ~ 2, squares).

To test this hypothesis, we first note that if flow is driven by a mass density gradient in the surrounding fluid, then we would expect the flow orientation to be sensitive to the direction of gravity. We therefore engineered a setup to measure the flow around the cluster in the same plane (the mid-plane of the cluster) in two opposing orientations of the setup with respect to gravity. In other words, we start with the cluster on the bottom of the chamber and then flip the chamber upside down so that the cluster is on the top of the chamber. In such a scenario, the direction of bouyancy-driven flows in the fixed plane of imaging should reverse when the orientation of the cluster is changed. This is precisely what we observed upon measuring the direction of the flow fields (Fig. 3A and movies S5 and S6), thus establishing that the observed flow is sensitive to gravity and driven by a density gradient in the surrounding fluid.

How are these fluid density gradients generated? As noted earlier, snowflake yeast metabolism relies on the uptake of nutrients (mostly glucose) from the surrounding fluid, with ethanol being produced as an outcome of fermentation. We thus placed clusters in media with and without the primary carbon source for metabolism (i.e., phosphate-buffered saline media with or without glucose), examined dead clusters in the presence of glucose (see the Supplementary Materials for more details and table S1), and checked for the presence of the flows. As a quantitative test for the presence of an advective flow, we tracked the motion of micron-sized tracer particles around snowflake yeast in different media conditions. In the absence of active fermentation (i.e., due to the yeast being dead or alive but in media lacking glucose) (Materials and Methods, Fig. 3B, table S1, and movies S7 to S9), the tracer particles exhibited diffusive Brownian motion, quantified by a linear relationship in the evolution of their mean-squared displacement (MSD) with time, i.e., MSD(*t*) ~ *t* (Fig. 3B and Materials and Methods). In contrast, around live clusters suspended in growth media, the MSD of the tracer particles exhibited a super-linear scaling with time; specifically, MSD(*t*) ~ *t*^2^, indicating that they were advected because of the presence of flows (Fig. 3B and movies S7 to S9). Further, we found that there is a threshold glucose concentration below which no flows were observed. This phenomenon was quantified by measuring MSD as a function of time and fitting to MSD ∝ t^α^ and extracting the best-fit power law exponent α (Fig. 3C and movies S10 to S12). A value of α ~ 2 is reflective of an advective environment, and the tracer motion is ballistic; on the other hand, when α ~ 1, tracer motion is diffusive. On the basis of these observations, we conclude that the metabolism of the snowflake yeast clusters drives a density-dependent mechanism to generate a circulatory flow in the ambient fluid.

If metabolism is sufficient to drive advective flows in macroscopic snowflake yeast, then why is it that we do not see these flows around all metabolically active organisms, regardless of their size? To determine whether microscopic snowflake yeast also generated flows or whether flows emerged only beyond a threshold size, we measured isolates from different time points of the MuLTEE. We found that isolates from early time points in the evolution experiment did not induce fluid flows—the tracer particles around the clusters of this size exhibit purely diffusive motion (Fig. 3D and movies S13 and S14). Only clusters from later in the evolution experiment (>300 transfers) were able to generate advective flows (Fig. 3D). By plotting the measured values of α against cluster size, we observe that α increases from ~1 to ~2 as a function of cluster size (Fig. 3D, Materials and Methods, table S2, and movies S13 and S14). This observation suggests that the metabolic activity of a sufficiently large cluster is necessary to generate these flows. To confirm if this is indeed the case, we fragmented macroscopic clusters (radii ≈ 1 mm) into smaller pieces. We found that while smaller fragments were unable to generate flows, fragments larger than the threshold size retained the capacity to generate flows (Fig. 3E, fig. S10, and movies S15 and S16). Together, metabolic activity of clusters beyond a threshold size, mediated by sufficient nutrients in the ambient medium, drives a spontaneous fluid density gradient that results in advective flows around the clusters.

**Fig. 4.**
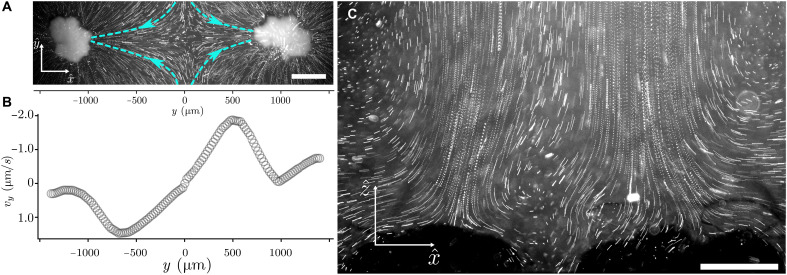
Snowflake yeast clusters act as individual metabolically powered density pumps. (**A**) Flows generated by the snowflake yeast are localized around individual clusters. The flows around two neighboring clusters exhibit a stagnation point between the clusters. Scale bar, 500 μm. (**B**) Velocity along the line connecting the two clusters shows a singular stagnation point of the flow. (**C**) Presence of such a stagnation point is also seen from the traces of the vertical plumes of two clusters that are within a cluster-radius distance from each other. Scale bar, 500 μm.

Last, while we have demonstrated that the observed advective flows arise due to metabolically driven buoyancy gradients, it is unclear if the low density fluid exhibits an instability outside of clusters. In other words, we asked whether clusters placed nearby each other would produce a single combined flow near the midpoint between them or if individual organisms each generate their own flows. We found that clusters, even those placed only a few cluster lengths apart, produced flow toward themselves (Fig. 4A and movies S17 and S18). This effect was quantified using particle imaging velocimetry to measure the time-averaged flow fields between two large clusters. The streamlines of the flow indicate a stagnation point between the clusters. This stagnation point and flow reversal on either side of the stagnation point can be seen from the quantification of the flow velocity along a line connecting the centers of two neighboring clusters (Fig. 4B). We found that the velocity dropped to zero roughly at the midpoint between the two clusters; further, the flow velocity pointed away from the center toward the clusters. This feature is seen even when the clusters are within a cluster distance of each other (Fig. 4C and movies S17 and S18). In sum, the flows are an emergent feature of single clusters, and therefore, each individual cluster acts as an independent metabolically powered density pump.

## DISCUSSION

Growth in three dimensions poses an inherent physical challenge for multicellular organisms: As they grow in size, their surface area to volume ratio decreases, which proportionally limits the surface available for the diffusive exchange of nutrients and waste products between the organism and its environment (*11*, *34*). Consequently, cells located in the interior of these large organisms may be deprived of nutrients, and therefore growth limited, due to the insufficient diffusive transport of resources (*49*). This limitation is thought to impose an upper bound on the size that multicellular organisms can achieve in the absence of evolved transport mechanisms, such as vascular systems, which efficiently distribute nutrients throughout the organism (*11*). Here, we demonstrated that large, undifferentiated multicellular organisms can overcome diffusion limitations through emergent metabolically driven flows. The flow emerges when the cells within clusters above a threshold size metabolize sufficiently quickly, creating a sustained density gradient in the surrounding liquid environment. Snowflake yeast are thus able to grow exponentially to macroscopic size, indicating that a consistent (i.e., size independent) proportion of the cells are reproducing as the cluster grows. It should be pointed out here that exponential growth per se may not be crucial for the evolution of multicellularity. Rather, the exponential growth we observe is evidence that diffusion is no longer limiting, i.e., nutrients and waste are being transported throughout the whole cluster. Overcoming diffusion limits is especially important for a multicellular organism to achieve large size, which is commonly believed to be a prerequisite for complex morphology.

The flows in our experiments are a consequence of yeast metabolism. There are precedents of metabolically created density driven flow: for example, single-celled (as opposed to snowflake) yeast colonies grown in a viscous medium stir their ambient media via a spontaneously driven baroclinic instability that is due to cellular metabolism (*45*). The flows in these experiments cause the yeast colonies to break up, as the unicellular yeast are not attached to one another, ultimately disrupting the flow. Convective flows have also been seen in growing bacterial populations (*46*), which later work demonstrated was rather due to evaporatively driven flows (*48*) or surface tension–driven (Marangoni) flows (*50*, *51*). On the other hand, bacterial biofilms have been shown to transport nutrients via structures that act like channels (*52*, *53*). While channels that pass through biofilms create more interfaces for diffusive flux (*53*), a more complex, fractal architecture of capillaries, such as that found in the circulatory systems of animals, is needed to ensure that nutrients can actually reach every cell (*54*). Despite this transport, biofilms have not been shown to exhibit exponential growth. In addition, biofilms typically have a dense extracellular matrix that also may prevent sufficient fluid transport through the interstices, especially where there are no channels. What we demonstrate in this paper is a much more facile mechanism that does not need to fulfill specific mathematical rules, and is independent of topologically complex flow channels. During the MuLTEE, snowflake yeast evolved to form mechanically durable clusters (as strong and tough as wood) via cellular entanglement (*30*, *31*). In addition to mechanical stability, the cellular entanglement ensures that the snowflake yeast are porous and therefore benefit, from the flows they generate, to grow up to macroscopic sizes. Thus, the structure and porous architecture of snowflake yeast, which is a consequence of the evolution during MuLTEE, may play an important role in exponential growth via spontaneous density-driven flows.

This work highlights the critical interplay between physical and biological processes in the evolution of multicellularity. The spontaneous emergence of flows in snowflake yeast clusters demonstrates how a purely physical mechanism, arising from the basic physical laws governing fluid dynamics, can profoundly affect the development of a biological system. These flows act as a “biophysical scaffold,” enabling a key trait—the ability to overcome diffusion limits—without requiring any dedicated structural adaptations. In the snowflake yeast model system, biophysical mechanisms have previously been shown to underpin key steps in the transition to multicellularity, such as the origin of a life cycle via packing-induced strain (*55*) and the emergence of heritable multicellular traits via a growth pattern guided by maximum entropy (*56*). Here, we extend these results to show that behaviors once thought to require sophisticated adaptations may instead arise “for free,” as a result of the emergent biophysics of simple multicellular systems. This suggests that the inherent physical properties of biological systems may have been crucial in enabling the evolution of unique multicellular traits by allowing access to phenotypes that can subsequently be refined and stabilized by selection. It will be interesting to examine how fluid dynamics affects the subsequent evolution of multicellularity in the snowflake yeast model system. Behaviors affecting flow may become genetically assimilated (*57*, *58*), and yeast evolved in static media may evolve multicellular morphological innovations that increase flow-based nutrient transport.

One intriguing question is why these flows are not observed in earlier generations of the MuLTEE. Our findings suggest that these buoyancy-driven flows become robust only once clusters surpass a key size threshold, enabling sufficient total metabolic output to sustain the necessary density gradients. Thus, nascent snowflake yeast lineages that fracture at a small size exhibit diffusive nutrient transport. In principle, other undifferentiated multicellular or large colonial microbes could exploit similar flows if they meet three main conditions: sufficiently large size, substantial metabolic activity and a structurally permeable morphology that allows fluid exchange throughout the colony. More generally, scaling arguments indicate that the metabolic rate needed to maintain a persistent flow depends on several organismal and environmental parameters: the intrinsic growth kinetics of cells, the effective viscosity of the fluid, diffusive fluxes of key nutrients, and the colony’s internal architecture.

The evolution of multicellularity has long been thought to be constrained by fundamental physical limitations, chief among them being the diffusive transport of nutrients, which becomes increasingly inadequate at larger organismal sizes (*9*, *56*). Strikingly, we found that an entangled morphology, which first evolved to provide mechanical stability to snowflake clusters growing in rapidly shaking media (*31*), has the serendipitous side effect of enabling snowflake yeast to grow large enough to spontaneously generate circulatory flows via a widespread biophysical mechanism—buoyant instabilities triggered by localized metabolism. This work demonstrates how trade-off breaking innovations can arise through the co-option of conserved biophysical mechanisms, with latent physical processes opening up frontiers of phenotypic evolution when harnessed by newly evolved traits. This observation fits into an emerging view that the interplay between physical and biological processes is both common and highly impactful in shaping evolutionary trajectories (*59*, *60*) and highlights the critical role of biophysical interactions in the origin of new levels of biological organization.

## MATERIALS AND METHODS

### Yeast strains

We used evolved isolates of snowflake yeast from anaerobic line 5 of the MuLTEE (*30*). For almost all experiments (exponential growth, flow fields, and metabolism assays), we used the 1000-day evolved strain from line 5 (PA5 t1000). For the cluster size experiments in Fig. 3, we used 200-, 400-, 800-, and 1000-day evolved strains from line 5 (PA5).

### Cell culture

The yeast were cultured in yeast extract peptone dextrose (YEPD) media (1% yeast extract, 2% peptone, and 2% dextrose) at 30°C in a shaking incubator at 250 rpm.

### Quantifying cluster area during growth

A single cluster was placed in liquid YEPD (see cell culture methods) in one well of a 12-well plate. A 45° mirror (Thorlabs, Right-Angle Prism Dielectric Mirror, 400 to 750 nm, *L* = 10.0 mm) was placed in the chamber next to the cluster, making the side profile of the cluster visible. Each of these clusters was imaged every 30 min for 12 hours on a Zeiss Axio Zoom.V16 microscope. Both the top of the cluster and the side of the cluster (via the mirror) were imaged at every time point. The clusters were kept at room temperature during this time. For analysis, clusters were segmented using the connected components algorithm in the scikit-image library with Python 3.10. The total number of pixels of the segmented clusters was found.

### Measuring flow fields

To measure flow fields, we filled a shallow well (WPI’s FluoroDish tissue culture dishes, with a well 1.2 mm in height and 23 mm in diameter) with YEPD (or other liquids, depending on conditions being tested) and placed one large snowflake yeast cluster in the well. The well was then covered with a cover slip, larger than the well diameter, to seal it. Images were taken at a rate of one image every 5 s on a Zeiss Axio Zoom.V16 microscope or Olympus XI81 inverted microscope. The presence and absence of advective tracks were quantified using particle tracking with the Mosaic plugin on the image analysis software ImageJ.

To measure plumes in the XZ plane, we used a 45° angle mirror (MRA12-E02, Thorlabs) placed in one well of a 12-well plate with 3 ml of YEPD media and a single (or multiple) clusters. Images were taken at a rate of at least 1 fps. The flow velocities in Fig. 2 were quantified using the TrackMate software in Fiji/ImageJ with a Laplacian of Gaussian (LoG) spot detector and a Kalman filter for tracking. The tracks were thresholded for quality, track length, and maximum speed to remove spurious tracks.

### Literature review for flow speeds

To generate Fig. 2C, we searched the literature for flow fields of ciliated or flagellated microorganisms and small multicellular organisms. We took the maximum flow speed, if provided in the text of the papers, or if not, we took the highest recorded speed from charts of the flow fields. For snowflake yeast, we took an estimate on the higher end of the vertical flow speeds as quantified by particle tracking (described above) in Fig. 2A.

### Gravitational flow field assay

To measure the effect of the axis of gravity on the orientation of the flow, we made a chambers using polydimethylsiloxane (PDMS), a flexible silicone polymer. These chambers were circular in shape with a diameter of ≈800 μm and a height of ≈500 μm. To adhere the cluster to the one side of the chamber, we used a mixture of 0.5% agar in phosphate-buffered saline. Just before the agar became stiff, we placed a single cluster of ≈300-μm radius atop the agar. Once the cluster was fully adhered (~1 to 2 min), we filled the chamber with YEPD nutrient media supplemented with 0.5-μm red fluorescent protein–coated polystyrene beads. We placed a cover slip atop the chamber and sealed it with nail polish. This allowed us to invert the entire chamber without dislodging the cluster.

### Protocol for breaking clusters

To break the clusters without damaging them, we used a cut pipette tip and pipetted a single cluster from the t1000 population (≈1000 μm). Via the mechanical action of the pipetting, we were able to break the single cluster into smaller clusters of ranging from ≈50 to ≈600 μm radius. We allowed the resulting liquid culture to settle under gravity for 40 s. This resulted in most of the larger clusters settling to the bottom of the tube. By pipetting from either the surface or the bottom of the liquid, we were able to recover small clusters of ≈50-μm radius and large clusters of ⪆300-μm radius.

## Supplementary Materials

### The PDF file includes:

Sections S1 to S7 Figs. S1 to S10 Tables S1 and S2 Legends for movies S1 to S27
