# Supplementary material for: Metabolically driven flows enable exponential growth in macroscopic multicellular yeast
Source: Sci Adv. 2025 Jun 20;11(25):eadr6399. doi: 10.1126/sciadv.adr6399 (PMC12180493; doi:10.1126/sciadv.adr6399)
Supplement: Supplementary file 1 — Sections S1 to S7 Figs. S1 to S10 Tables S1 and S2 Legends for movies S1 to S27 [file sciadv.adr6399_sm.pdf]

Science Advances
AAAS

Supplementary Materials for

Metabolically driven flows enable exponential growth in macroscopic multicellular yeast

Nishant Narayanasamy *et al.*

Corresponding author: Shashi Thutupalli, shashi@ncbs.res.in; Peter Yunker, peter.yunker@gatech.edu

Sci. Adv. **11**, eadr6399 (2025)
DOI: 10.1126/sciadv.adr6399

The PDF file includes:

Sections S1 to S7
Figs. S1 to S10
Tables S1 and S2
Legends for movies S1 to S27

Other Supplementary Material for this manuscript includes the following:

Movies S1 to S27

This document is organized in the same order as the figures presented in the main text of the paper. The material includes detailed protocols, data acquisition and analysis methods for the data shown in each of the panels. This is followed by the Supplementary Figures, Tables and information about Supplementary Movies.

S1. Description for Data in Figure 1 and associated Supplementary Figures

S1.1 Figure 1 A

Snowflake yeast clusters from PA5 t1000 were grown in YEPD medium until stationary phase, harvested and washed in double distilled water three times. The clusters were next diluted in increasing concentrations of ethanol until final concentrations of 100 percent ethanol was reached. Clusters were super-critically dried in a Leica EM CPD300 Critical Point Dryer. Once dried, the clusters were sputter coated with gold at 20m amps for 90 seconds to achieve a gold coating of $\approx$5 nm. Finally, clusters were imaged on a Zeiss Merlin compact electron microscope. No processing was done to the resultant images.

S1.2 Figure 1 B and SI Figs. S1, S2, S3, S4

Two different media conditions were tested — YEPD in 2% agar and YEPD in water. In order to prepare YEPD agar plates, yeast extract (1%), peptone (2%) and agar (2%) were dissolved in 475 ml distilled deionized water and sterilized by autoclaving. Following this, 25 ml of 40% filter sterilized glucose was added to achieve a final glucose concentration of 2%. 3ml of this medium was poured into individual 35 mm Petri dishes and left to set for 30 minutes. Once set, in order to ensure as little fluid on top of the agar as possible, the plates were further allowed to dry for 2 hours prior to usage.

Preparation of liquid YEPD medium was exactly the same as mentioned for agar without

the addition of 2% agar. Before pouring 3 ml of the liquid YEPD into a petri dish, a thin layer (1 ml) of 0.5% agar in phosphate buffered saline was poured into each 35 mm Petri dish. This layer of 0.5% agar in phosphate buffered saline served as a pad to prevent the clusters from moving once inoculated, and since it does not contain any nutrition, it does not affect the interpretation of our results.

Plates containing either liquid YEPD or YEPD agar were inoculated with single clusters which had been isolated and washed thrice in PBS following growth in YEPD for $\approx$24 hours. For single cell experiments, similar preparatory steps were taken with the only difference being that, following the last wash, cells were pelleted and 2 μl of the pellet was used to inoculate each YEPD agar plate. (Single cell experiments could not be performed in the same way as those performed for clusters in liquid YEPD, as single cells would diffuse, preventing accurate imaging of the sample.)

Plates were next placed on an Epson v800 flatbed scanner and scanned every ten minutes for 10 hours at a resolution of 1200 dpi. The ambient temperature and relative humidity of the room in which imaging was done was 25°C and $< 60\%$.

S1.3 Figure 1 C and SI Figs. S5, S6, S7

PA5 t1000 clusters were grown for 24 hours in 10 ml of YEPD in a shaking incubator at 30°C and 250 RPM. Single large clusters were obtained for the experiments described in this section by taking 1 ml out of this 24 hour culture, letting it settle for around 30 seconds, and pipetting out most of the supernatant to remove smaller clusters, and pipetting in fresh YEPD. This washing was repeated several times to ensure the removal of any small clusters. Then around 500 ml of this mixture was pipetted into an empty Petri dish with a few ml of YEPD in order to spread out the large clusters into a larger surface area so that individual clusters would be visible to the eye and available to select for experiments. A chosen large cluster is carefully pipetted

up into a wider-bore tip 1000 μl pipette tip, and this tip is gently placed into fluid. The cluster is allowed to sink down the pipette and out into the waiting fluid (without actively pipetting it out).

A single PA5 t1000 yeast cluster was placed in 3 mL of liquid YEPD in one well of a 12-well plate. A 45-degree mirror (Thor labs right-angle Prism Dielectric Mirror, 400-750 nm, L = 10.0 mm) was placed a few millimeters from the cluster. Images were taken of the top of the cluster and the side of the cluster (via the mirror) every 30 minutes for 12 hours using a Zeiss AxioZoom.V16 microscope. Three replicate measurements were obtained.

The clusters were segmented by binarizing and using a connected components algorithm in the scikit-image Python package. The estimated volume was calculated by multiplying the area of the topview (calculated as the number of pixels multiplied by the area scale factor) by the average height of the sideview (calculated by finding the height in pixels of each column from top to bottom, averaging, and multiplying by the distance scale factor). SI Fig. **??** compares this estimate to two other estimates: the cube of the maximum radius of the sideview, and the cube of the maximum radius of the topview.

S2. Description for Data in Figure 2 and associated Supplementary Figures

S2.1 Figure 2 A, B, and SI Figs. S8, S9

PA5 t1000 clusters were grown overnight in YEPD media at 30°C. To image a cluster, the cluster was placed in a small Petri dish with a 45-degree mirror (Thor labs right-Angle Prism Dielectric Mirror, 400-750 nm, L = 10.0 mm), and images were taken on a Zeiss AxioZoom.V16 microscope at approximately 3 fps for both the top view of the cluster and the side view in the mirror.

Particle tracking was done using the TrackMate plugin in FIJI with a LoG spot detector and a Kalman filter for connecting spots. Exact parameters can be found in the supplemental data. The tracks were filtered for spot quality, track length, and max speed to remove as many spurious tracks as possible. Tracks were overlaid and visually compared to the video to check that they were capturing the motion of the tracing particles.

A composite image of the video was made by taking the max of every fourth frame, creating an effect similar to a star tracks image in order to show the particle movement. The particle tracks were then overlaid on the right side of the image. The colorbar indicates the maximum speed of each track.

See Figs. S8 and S9 and corresponding Supplementary Movies for additional examples of particle tracking of flows.

S2.2 Figure 2 C

A literature search was performed for flow fields of small organisms, especially ciliated and flagellated organisms. Most reports found included a chart displaying the flow field around the organism, from which we obtained the approximate maximum speeds of the flow fields. The value used for snowflake yeast is the approximate maximum value from A. The flow speeds

found around snowflake yeast clusters can vary based on the cluster, the chamber geometry, and other factors.

S2.3 Figure 2 D

Single snowflake yeast clusters were assayed across 10 hours for the presence or absence of ambient fluid mixing in pseudo 2D chambers. These chambers were prepared by sticking multiple layers of double sided tape, with each tape having a height of ≈0.05 mm (chamber heights were adjusted depending on the size of the clusters being observed, ranging from 0.1 mm - 0.6 mm) on a previously cleaned glass slide. PA5 t1000 clusters were grown for 2 hours in YEPD liquid medium, following which they were washed thrice in phosphate buffered saline and inoculated into the chamber along with YEPD liquid supplemented with 0.5 μm GFP coated beads. Presence or absence of advective mixing was assayed every hour by taking a 2-minute video at a frame capture rate of 5 fps in the GFP fluorescence channel.

The video generated was loaded into image analysis software Fiji, where, following conversion to 8-bit grayscale, particles were detected and traced using the Mosaic particle tracker plugin. Detected particles were then analyzed in Matlab to calculate the mean squared displacement (MSD) of individual particles across each time step. These MSDs were then plotted against the time step in order to quantify the presence or absence of ambient fluid mixing. A slope of ≈ 1 on a log log MSD vs. time step plot corresponds with a diffusive fluid environment and a slope of ≈ 1 corresponds with a advective fluid environment. The velocity of the mixing is the intercept of this graph and was plotted against the time points. The velocity was normalized since the magnitude of the mixing is dependent on the geometry of the chamber and varies depending on the imaging condition.

S3. Description for Data in Figure 3 and associated Supplementary Figures

S3.1 Figure 3 A

A chamber was engineered that could be inverted completely without any leaks, and more importantly, without clusters to dislodging from the the position in which they were inoculated. In order to make the chamber, a silicone polymer known as poly dimethyl siloxane, PDMS for short, was used. This polymer, when treated with the appropriate curing agent, hardens into a flexible and transparent r ubber. Approximately 10 ml of this polymer was allowed to cure in a 60 mm Petri dish, which resulted in a circular rubber of 60 mm radius and ≈5 mm height. Once cured, individual square shaped pieces of ≈10 mm side length were cut which were then punched with a 5 mm biopsy punch in order to create a circular hole with 5 mm radius and 5 mm height. Next the cut piece of PDMS and a previously cleaned coverslip were plasma cleaned on one side. Once plasma cleaned, the piece of PDMS was bonded onto the coverslip, resulting in a circular well of 5 mm radius and 5 mm height. Into this well $200\ \mu$l of 0.5% agar in PBS was added in order to adhere clusters to one side of the chamber. Within 10 seconds of the addition of the base agar, a single PA5 t1000 cluster was inoculated atop the base agar. The chamber was next filled with liquid YEPD supplemented with 2% glucose or PBS supplemented with 2% glucose (roughly $500\ \mu$l) and $0.5\ \mu$m GFP tracer particles. Once full, another previously cleaned 20 mm coverslip was placed on the open side of the well and secured with clear nail polish. This setup was left to dry for 10 minutes. Sealing the chamber with nail polish was done to ensure that no evaporative flows were g enerated. Imaging was done on this setup both with its right side up and also with the upside down. We confirmed that the cluster had not dislodged from the point at which it had been adhered by checking the change in the z-focus position. Imaging was done at both the first plane at which the cluster came into focus (bottom) and the

last plane after which the cluster was out of the focal plane (top). The microscope used was a IX81 Olympus Epifluorescence microscope. Imaging was done at 4X magnification in the GFP fluorescence channel at a frame capture rate of 5 fps.

S3.2 Figure 3 B

Different media conditions were assayed to check for presence or absence of flows. Pseudo 2D chambers were prepared by sticking multiple layers of double sided tape, with each tape having a height of ≈0.05mm (chamber heights were adjusted depending on the size of the clusters being observed, ranging from 0.1 mm - 0.6 mm) on a previously cleaned glass slide. Once the chambers were prepared, Snowflake yeast clusters (anaerobic line 5 t1000) grown for 24 hours in YEPD were harvested and washed three times in PBS, following which they were transferred to a vial containing the medium being tested supplemented with $0.5\ \mu\mathrm{m}$ GFP fluorescent beads (available in table). Individual clusters were taken from the vial containing the medium being tested and placed into a chamber. Once inoculated, a coverslip (20 X 20 mm) was placed on top of the chamber, this was then sealed with transparent nail polish which was allowed to dry for ≈10 minutes. Sealing the chamber with nail polish was done to ensure that no evaporative flows were generated. Imaging was done at magnifications of 10X and 20X in the GFP fluorescence channel at a frame capture rate of 5 fps.

S3.3 Figure 3 C

Presence or absence of flows was assayed as detailed above for different concentrations of glucose in phosphate buffered saline.

S3.4 Figure 3 D

We used various time points from one of the experimental evolution lines (available in table) and assayed them for the presence or absence of ambient fluid mixing (as mentioned in SI for

Fig. 3 B). We only used one media condition for this experiment (PBS supplemented with 2% glucose). Chamber heights were adjusted depending on the size of the cluster being assayed.

S3.5 Figure 3 E and SI Fig. S10

An experiment was devised where PA5 t1000 clusters ($>$ 1 mm radius) were pipetted with a cut tip to generate different sized clusters. Clusters were independently assayed for the presence or absence of ambient fluid mixing (as described in SI for Fig. 3 B).

Experiments were performed with the ancestral population from the anaerobic line 5 (which had on average radii smaller than the threshold radii) were grown as colonies on YEPD 2% agar plates, following which a piece of the colony greater than the threshold size was picked and assayed for the presence or absence of ambient fluid mixing (as described in SI Fig. **??**). Similar experiments were also conducted for single cell colonies.

S4. Description for Data in Figure 4 and associated Supplementary Figures

Multiple snowflake yeast clusters from anaerobic line 5 t1000 were observed to understand how ambient fluid mixing is affected by the presence of more than one cluster. Growing and washing steps for the clusters are exactly as mentioned in Fig. 2 D. Observations were made in 35 mm Petri dishes with 1 ml of 0.5% agar in PBS to adhere clusters to the point of inoculation. Following adhering, 3 ml of YEPD liquid supplemented with 2 μm GFP coated particles were added. To prevent Marangoni flows due to evaporation 2 ml of squalene was added atop the fluid layer. This oil floats atop water and is biocompatible, hence it doesn't interfere with our interpretation of the results. The setup was imaged on a Leica stereo microscope in the GFP fluorescence channel at a magnification of 7.9X with a frame capture rate of 0.1 fps.

S5. Supplementary Figures

S5.1 Associated with Figure 1

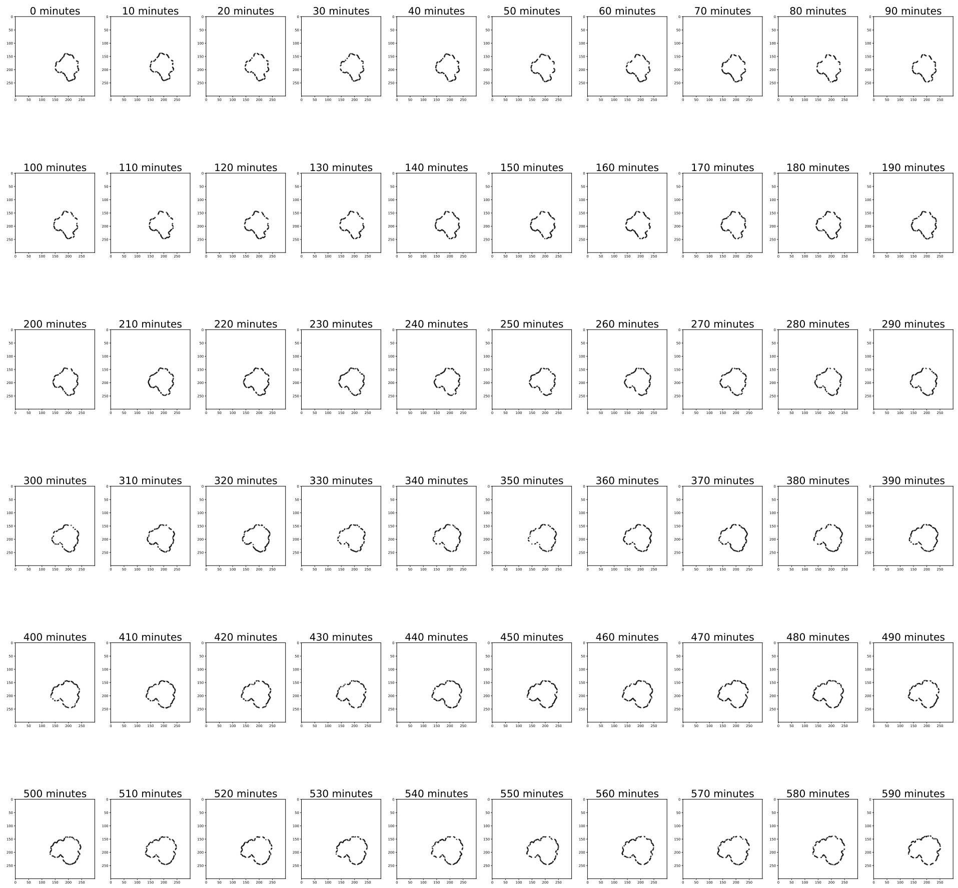

Fig. S1: **Growth of snowflake yeast over 12 hours.** Grown in YEPD 2% Agar. Viewed from the top.

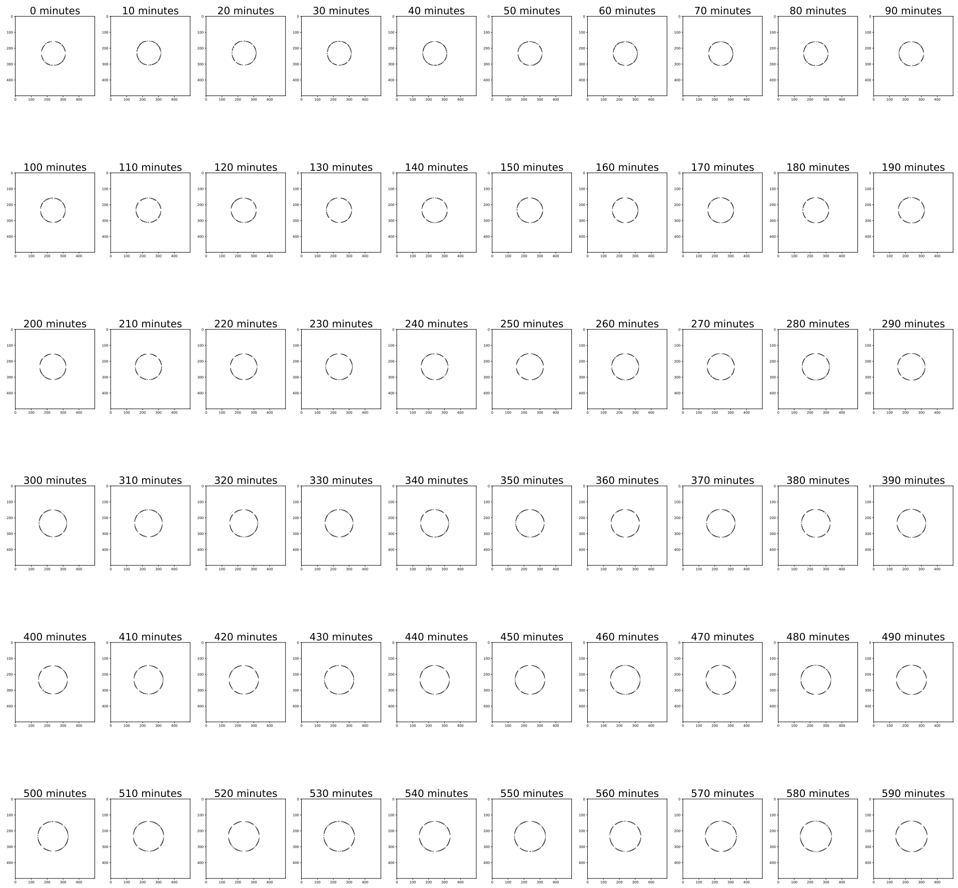

Fig. S2: **Growth of single-celled yeast over 12 hours.** Grown in YEPD 2 % Agar. Viewed from the top.

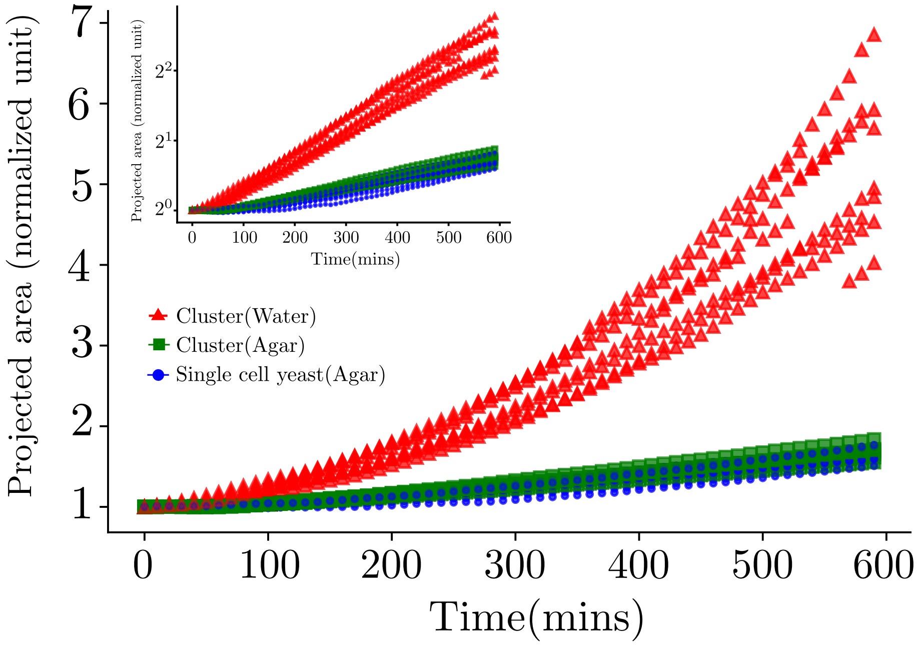

Fig. S3: **Comparison of growth of snowflake clusters in YEPD (liquid) and YEPD (agar) and single-celled clusters in agar.** Clusters grow exponentially in Liquid YEPD, a medium that supports flows, and they grow sub-exponentially on 2% agar, a medium that does not support flows.

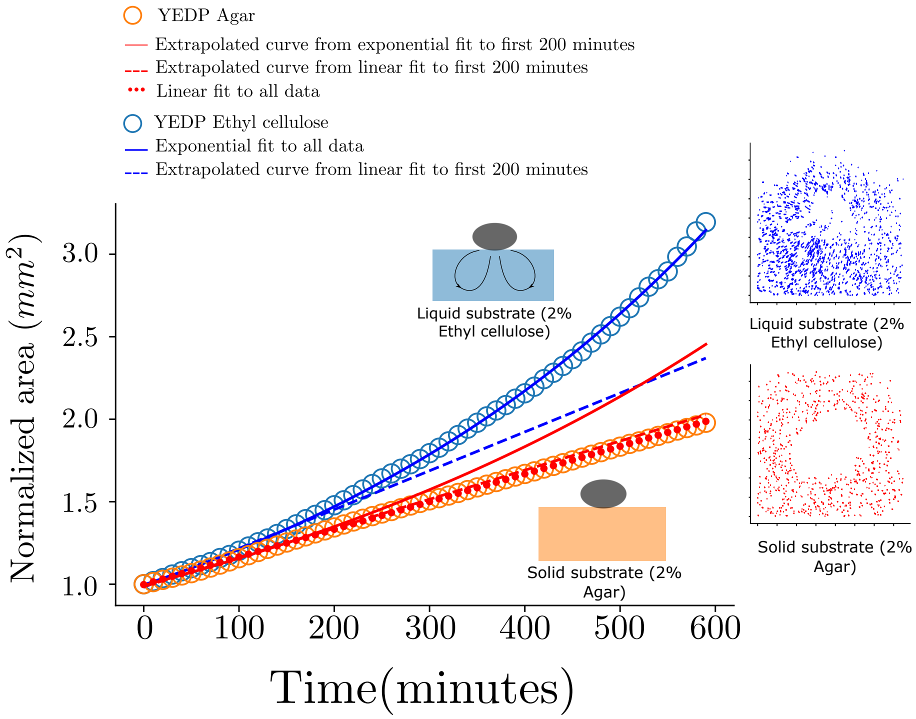

Fig. S4: **Comparison of growth of snowflake clusters on top of YEPD (2% agar) and YEPD 2% ethyl cellulose.** Clusters grow exponentially on top of ethyl cellulose, a medium that supports flows, and they grow sub-exponentially on 2% agar, a medium that does not support flows. This provides evidence that flows enable exponential growth.

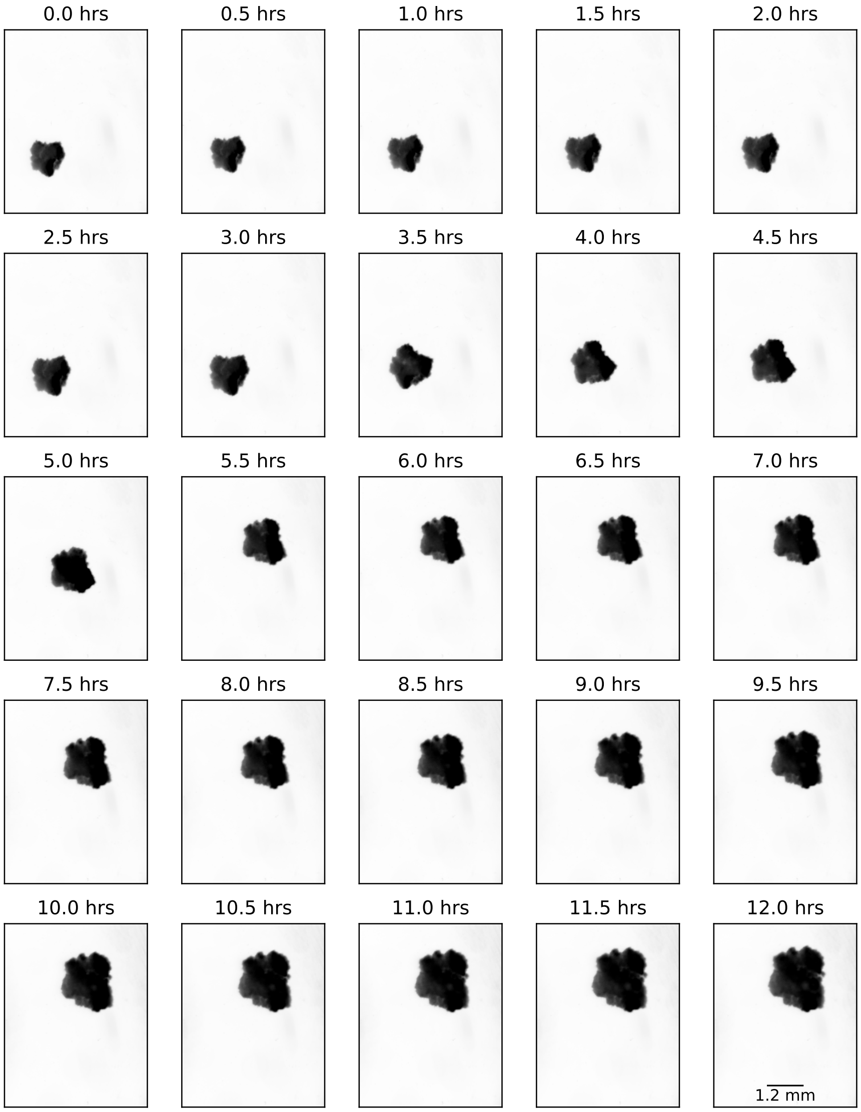

Fig. S5: **Images of growth of a snowflake yeast cluster over 12 hours.** Grown in liquid YEPD. Viewed from the top.

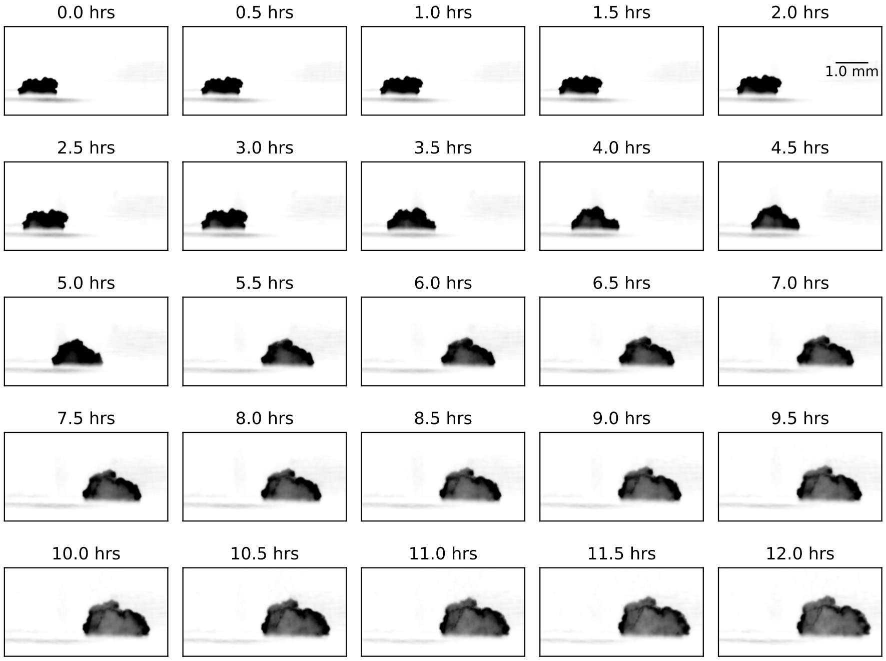

Fig. S6: **Images of growth of a snowflake yeast cluster over 12 hours.** Grown in liquid YEPD. Viewed from the side with a 45-degree angle mirror.

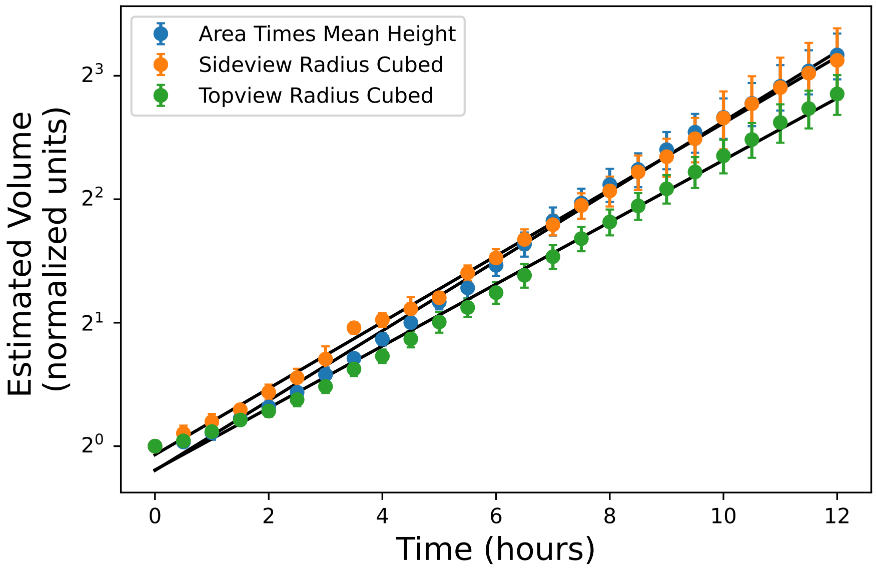

Fig. S7: **Three different volume estimates for the growth of snowflake yeast clusters.** The image data in fig. S1-S2 was used to make three different volume estimates, as shown in the legend. The 'area times mean height' estimate was used in the main text, but the others produce similar results.

S5.2 Associated with Figure 2

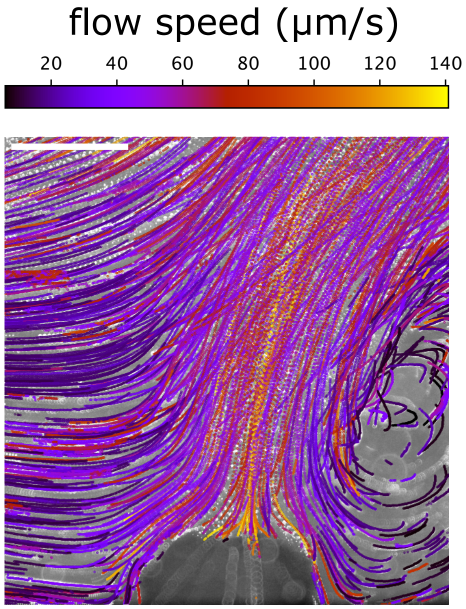

Fig. S8: **Additional example of particle tracking for the X-Z view of a cluster (viewed with a 45-degree mirror).** See movie S19. Scale bar: 500 μm.

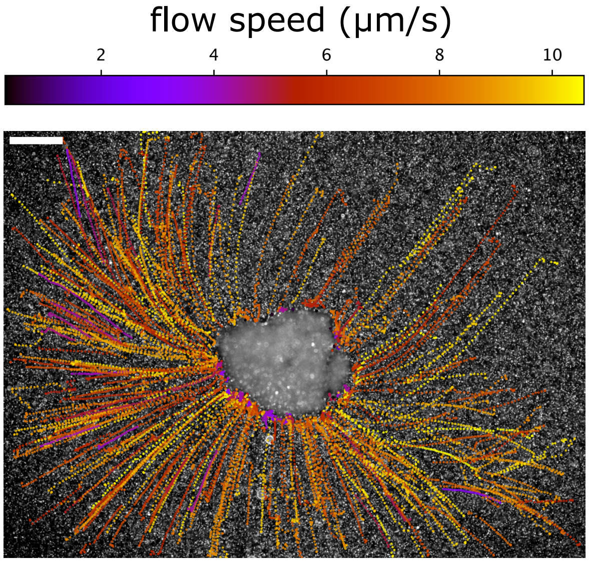

Fig. S9: **Additional example of particle tracking for the X-Y view of a cluster.** See movie S20. Scale bar: 500 μm.

S5.3 Associated with Figure 3

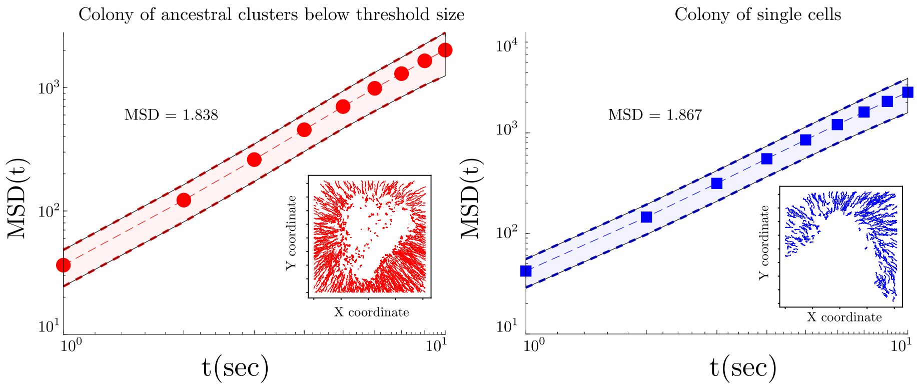

Fig. S10: **Presence of flows in anaerobic ancestor (left) and single celled (CEN.PK) (right) when grown as colonies to increase effective size.** This provides evidence that flows are generated when there is a large enough group of cells metabolizing together.

S6. Supplementary Tables

Table S1: **MSD slopes for different media conditions.** MSD slopes in different conditions highlight the importance of metabolism in generating fluid flows.

Media	MSD slope	Replicates
YEPD (live)	1.739	3
PBS + glucose (live)	1.840	3
PBS (live)	1.004	3
PBS + glucose (dead)	1.003	3

Table S2: **Cluster radii in μm and MSD slope.** MSD slopes for different cluster sizes indicate a threshold radius at which fluid flows emerge.

Radii	MSD
913.1073486	1.9762
225.895301	0.9736
139.3004523	0.9378
137.1524991	0.92
482.9431768	1.8957
737.078953	1.7217
364.827388	1.9441
169.6425654	0.9447
166.0494746	0.9504
190.9950942	0.9584
795.3747	1.9524
566.9491	1.9329
115.9202	1.0125
240.741	1.0031
337.4352	1.0343
302.4897	1.0236
721.4265	1.8544
494.6274	1.8831
362.1291	1.8905

S7. Supplementary Movie Information

S7.1 Associated with Figure 1

Movie S1: **Growth of snowflake yeast clusters on YEPD 2% a gar.** This is the movie that corresponds to Fig. 1B.

Movie S2: **Growth of snowflake yeast clusters in YEPD.** This is the movie that corresponds to Fig. 1B.

Movie S3: **Growth of CEN.PK colony on YEPD 2% agar.** This is the movie that corresponds to Fig. S2.

S7.2 Associated with Figure 2

Movie S4: **Flow in the vertical (X-Z) plane**. Viewed with a 45-degree mirror. This is the movie that corresponds to Fig. 2A.

Movie S5: **Flow in the horizontal (X-Y) plane.** This is the movie that corresponds to Fig. 2B.

Movie S6: **Flow in the horizontal (X-Y) plane at the start of time series measurement.** This is the movie that corresponds to Fig. 2C.

Movie S7: **Flow in the horizontal (X-Y) plane at the end of time series measurement.** This is the movie that corresponds to Fig. 2C.

S7.3 Associated with Figure 3

Movie S8: **Flow in the horizontal (X-Y plane) for a cluster that is adhered to the bottom of the chamber.** This is the movie that corresponds to Fig. 3A(bottom).

Movie S9: **Flow in the horizontal (X-Y plane) for a cluster that is adhered to the top of the chamber.** This is the movie that corresponds to Fig. 3A(top).

Movie S10: **Flow in the horizontal (X-Y) plane when live in the presence of PBS and 2% glucose.** This is the movie that corresponds to Fig. 3B (red).

Movie S11: **Flow in the horizontal (X-Y) plane when dead in the presence PBS and 2% glucose.** This is the movie that corresponds to Fig. 3B (blue).

Movie S12: **Flow in the horizontal (X-Y) plane when live in the presence of PBS.** This is the movie that corresponds to Fig. 3B (green).

Movie S13: **Flow in the horizontal plane (X-Y) when live in the presence of PBS and 0.002% glucose.** This is the movie that corresponds to Fig. 3C.

Movie S14: **Flow in the horizontal plane (X-Y) when live in the presence of PBS and 0.02 % glucose.** This is the movie that corresponds to Fig. 3C.

Movie S15: **Flow in the horizontal plane (X-Y) when live in the presence of PBS and 2% glucose.** This is the movie that corresponds to Fig. 3C.

Movie S16: **Flow in the horizontal plane (X-Y) for a cluster above the threshold size.** This is the movie that corresponds to Fig. 3D.

Movie S17: **Flow in the horizontal plane (X-Y) for a cluster below the threshold size.** This is the movie that corresponds to Fig. 3D.

Movie S18: **Flow in the horizontal plane (X-Y) for a cluster broken to above threshold size.** This is the movie that corresponds to Fig. 3E.

Movie S19: **Flow in the horizontal plane (X-Y) for a cluster broken to below threshold size.** This is the movie that corresponds to Fig. 3E.

Movie S20: **Flow in the horizontal plane (X-Y) for anaerobic ancestral clusters when grown as a colony to increase effective size.** This is the movie that corresponds to Fig. S10 (red).

Movie S21: **Flow in the horizontal plane (X-Y) for single cell yeast when grown as a colony to increase effective size.** This is the movie that corresponds to Fig. S10 (blue).

S7.4 Associated with Figure 4

Movie S22: **Flow in the horizontal (X-Y) plane for two clusters.** This is the movie that corresponds to Fig. 4A.

Movie S23: **Flow in the vertical (X-Z) plane for two clusters that are within a cluster-distance of each other.**

S7.5 Additional Examples

Movie S24: **An additional example of flow in the vertical (X-Z) plane**. Viewed with a 45-degree mirror. This is the movie that corresponds to Fig. S8.

Movie S25: **An additional example of flow towards the cluster in the horizontal (X-Y) plane**. This video was taken in a focal plane near the bottom of the cluster and shows the flow moving towards the cluster. This is the movie that corresponds to Fig. S9.

Movie S26: **An additional example of flow away from the cluster in the horizontal (X-Y) plane**. This video was taken in a focal plane near the top of the cluster and shows the flow moving away from the cluster.

Movie S27: **Growth of snowflake yeast clusters on YEPD 2% ethyl cellulose**. This is the movie that corresponds to Fig. S4.
